# Quality, Thermo-Rheology, and Microstructure Characteristics of Cubic Fat Substituted Pork Patties with Composite Emulsion Gel Composed of Konjac Glucomannan and Soy Protein Isolate

**DOI:** 10.3390/gels10020111

**Published:** 2024-01-31

**Authors:** Lai Wei, Yuqing Ren, Lu Huang, Xinnan Ye, He Li, Jian Li, Jinnuo Cao, Xinqi Liu

**Affiliations:** 1 National Soybean Processing Industry Technology Innovation Center, Beijing Technology and Business University, Beijing 100048, China; weilai00109@163.com (L.W.); ryq512@163.com (Y.R.); huanglulu1119@163.com (L.H.); 13611266368@163.com (X.Y.); liuxinqi@btbu.edu.cn (X.L.); 2 Key Laboratory of Green and Low-Carbon Pocessing Technology for Plant-Based Food of China National Light Industry Council, Beijing Technology and Business University, Beijing 100048, China; 3 Puluting (Hebei) Protein Biotechnology Research Limited Company, Handan 056000, China; jinnuocao@163.com

**Keywords:** plant-based, fat mimetics, pork batter, temperature scanning, sensory evaluation

## Abstract

Composite emulsion gel can effectively mimic animal adipose tissue. In this study, composite emulsion gels composed of soy protein isolates and konjac glucomannan (KGM) were prepared as plant-based cubic fat substitutes (CFS). The effects of CFS on the quality and structure of pork patties were investigated in terms of the proximate composition, lipid oxidation stability, technological characteristics, color, sensory attributes, texture, thermo-rheological behavior, and microstructure. CFS samples composed of various ratios of KGM were added to lean meat patties to ascertain the optimal CFS composition for its potential replacement of pork back fat in patties. The addition of CFS containing 7.0% KGM was found to decrease the hardness of the lean meat patties by 71.98% while simultaneously improving their sensory quality. The replacement of pork back fat with CFS also reduced the fat content of the patties to as little as 3.65%. Furthermore, the addition of CFS enhanced the technological characteristics, lipid oxidation stability, and surface color of the fat-replaced patties, with no significant impact on their overall acceptability. The gel network of the patties was shown to be fine and remained compact as the fat replacement ratio increased to 75%, while the texture parameters, storage modulus, and fractal dimension all increased. Quality and structure improvements may allow the composite emulsion gels to replace fat in pork patties to support a healthy diet. This study may be beneficial for the application and development of plant-based cubic fat substitutes.

## 1. Introduction

Typically, the fat content of animal meat products ranges from 15% to 35%, and concerns have been raised about the health implications associated with the consumption of such high-fat products [1]. The World Health Organization, for example, recommends that the daily energy intake from dietary fats should be controlled within a maximum of 30% of total energy intake [2], and the long-term excessive intake of long-chain saturated fatty acids and cholesterol from animal fats is commonly associated with serious metabolic diseases, such as cardiovascular diseases [3]. Consequently, the concept of a low-fat and healthy diet is gaining popularity. Animal fats do, however, play a crucial role in the processing of meat products, contributing positively to stability, technological characteristics, texture, and sensory attributes [4]. So, fat substitutes were employed to replace animal fat used in meat products, reducing the fat content without compromising the products’ quality.

Traditional fat substitutes, which include cereal flour-, dietary fiber-, and protein-based substitutes and pre-emulsification systems, do not sufficiently meet the call for solid-like structural adipose tissue [5]. Emulsion gels and oleogels possess semi-solid properties similar to animal adipose tissue and can be applied as a novel fat substitute to reduce fat content and/or improve fatty acid profiles in meat products such as patties and sausages [5]. When used as a fat substitute, emulsion gel exhibits excellent fat-reduction effects and oxidative stability, and it is also easy to prepare [6]. Animal fat components are part of the cellular structure and embedded within connective tissues, primarily composed of a protein network of collagen [7]. These characteristics contribute to the unique quality of meat products, providing them with specific visible textures and a distinctive appearance. However, the high viscosity and soft texture of emulsion gels restrict their formation into three-dimensional cubes [8]. To address this problem, konjac glucomannan (KGM) was employed to enhance gel strength and create a soy protein isolate (SPI)–KGM composite emulsion gel. This novel and feasible strategy was found to improve the texture and rheological properties of emulsion gels, enabling the development of three-dimensional, plant-based cubic fat substitutes (CFS) [9].

Fat replacement levels are limited by the influence of fat substitutes on the quality of a meat product, and different fat substitutes tend to exert various effects [6,10]. A composite emulsion gel, designed to mimic the characteristics of pork back fat, was developed using a blend of protein and polysaccharide [11]. Compared with the gel formed by only protein or polysaccharide, the sensory properties of the complex are closer to those of real fat. Yet the effects of replacing fat with this composite emulsion gel in pork patties have not been fully explored. Thus, in this work, the influence of CFS fabricated with KGM and SPI on the quality and structure of pork patties was investigated in terms of the proximate composition, lipid oxidation stability, technological characteristics, color, sensory attributes, texture, thermo-rheological behavior, and microstructure.

## 2. Results and Discussion

### 2.1. Effects of CFS Containing Different Concentrations of KGM on Lean Meat Patties

#### 2.1.1. Sensory Attributes

To determine the optimal KGM addition in CFS, konjac emulsion gels (KEGs) with various KGM concentrations (0–10.5%) were incorporated into lean meat patties. Reformulated meat products must possess acceptable sensory characteristics, which include appearance, flavor, juiciness, and tenderness [12]. The sensory evaluation results (Table 1) indicated that the concentration of KGM in the CFS significantly (*p* < 0.05) affected the appearance, aroma, juiciness, tenderness, and overall acceptability of the cooked lean meat patties. The appearances of totally lean meat patty (TLP), KEG3.5, and KEG7 were regarded as acceptable (score > 5), while those of KEG0 and KEG10.5 were not. A distinct and discernible milky-white fat analog was only observed in the meat patties with CFS containing 7.0% KGM or higher concentrations. However, the texture of the patty with the CFS containing 10.5% KGM was found to be firm and non-deformable, but clearly different from animal adipose tissue in terms of appearance and mechanical properties. KEG7 scored best in terms of appearance. The aroma scores of KEG0, KEG3.5, and KEG7 were significantly (*p* < 0.05) higher compared to the TLP, possibly due to the heat oxidation reaction of coconut oil in the CFS. Similarly, the three groups of patties with CFS received higher scores in terms of tenderness, while KEG10.5 was, again, an exception. The improvement in tenderness might have been due to the absorption of water and filling of the protein network by the KGM–SPI composite, which homogenized with the meat matrix, thereby influencing the thermal gel properties formed by myofibrillar protein [13]. The excessively compact structure of the CFS could not only prevent it from mixing with the meat matrix but might also block the release of volatile compounds, thus incapacitating its ability to improve tenderness and aroma. For juiciness, KEG0 received the highest score of 7.5, followed by KEG7 at 6.5. However, the addition of KGM to the CFS was found to have a negative effect on juiciness, as KGM restricts water movement and reduces the free water content of the CFS [14]. In terms of overall acceptability, the highest score was achieved by KEG7. These results demonstrated that the CFS containing 7.0% KGM most improved the sensory quality of the lean meat patties. Thus, this formula was selected for the replacement of pork back fat in subsequent experiments.

#### 2.1.2. Texture Profile

The characterization of a food’s mechanical properties using texture profile analysis plays an important role in understanding its texture. As shown in Table 1, the four textural parameters of the cooked lean meat patties with added CFS were significantly (*p* < 0.05) lower than those of the TLP. With the increase in KGM content in the CFS, the hardness of the meat patties initially decreased and then increased. KEG7 showed the lowest hardness (25.00 N), accounting for 28% of TLP, indicating that a proper KGM addition in CFS could decrease the mechanical strength of patties. This phenomenon is similar to pork back fat, which would lead to the formation of a looser meat–protein matrix network, resulting in a decrease in hardness [15] Adding KGM to the CFS would enhance their solid properties [11], and solid-like CFS may prevent the formation of an excessively compact meat–protein matrix network by physical obstruction in space. The springiness, cohesiveness, and chewiness of KEG3.5, KEG7, and KEG10.5 were similar to each other but significantly (*p* < 0.05) lower than those characteristics in the patties without KGM. These results demonstrated that the addition of CFS and KGM might help form a looser meat–protein thermal gel, thus decreasing the hardness of cooked patties.

### 2.2. Effect of Replacing Pork Back Fat with CFS

#### 2.2.1. Proximate Composition

The selected KEG was used as CFS to replace 25–100% (CFS25–CFS100) pork back fat in meat patties. The patties without the replacement were set as the control group (CON). The use of CFS to replace pork back fat in the meat patties significantly (*p* < 0.05) affected the moisture content, protein content, and fat content of the raw meat patties (Table 2). As the fat replacement ratio increased, so too did the moisture and protein content of the meat patties, with CFS100 exhibiting the highest values of 73.13% and 18.41%, respectively. The increase in protein content was attributed to the presence of the SPI in the CFS. Also, the emulsion gels stabilized by gelatin increased the protein content in the patties [16]. As expected, the replacement of pork back fat with CFS led to a reduction in fat content. The fat content in CFS100 was the lowest (3.65%), which is a reduction of 82.65% compared to the CON. Furthermore, a significant amount of water was captured by the CFS, enabling a low proportion of fat in the patty and showing the suitability of CFS as an ideal substitute for reducing fat content. According to Regulation (EC) No. 1924/2006 for nutrition claims, meat patties can be labeled as having ‘reduced fat content’ provided more than 50% of pork back fat is replaced with CFS. In this study, the fat content of patties was reduced by more than 30% and reached 37.78%, 66.87%, and 82.65%, respectively. In general, the complete replacement of pork back fat with CFS was found to be the most satisfying in terms of proximate composition because these patties had the lowest fat content with the highest protein content and could be legitimately labeled as having ‘reduced fat content’.

#### 2.2.2. Lipid Oxidation

Lipid oxidation is a primary non-microbial factor leading to deterioration in the quality of meat and meat products [17]. The thiobarbituric acid reactive substances (TBARS) value can be used to quantify the levels of secondary products produced by lipid oxidation. The results are expressed as equivalents of malondialdehyde (mg MDA/kg), with a higher TBARS value indicating a higher degree of fat oxidation and more severe deterioration in meat quality [18]. The TBARS values of the raw meat patties both before and after 7 days of storage are presented in Table 2. No significant (*p* < 0.05) differences were observed in the TBARS values of the samples before storage; however, after 7 days of storage, the TBARS values for all samples significantly (*p* < 0.05) increased. Patties in which the pork back fat was replaced with CFS had comparatively significantly (*p* < 0.05) lower TBARS values, while the TBARS value of the control group was highest, reaching 0.38 mg MDA/kg. With the increase in the fat replacement ratio, the TBARS values of the patties significantly decreased after storage. Among them, CFS100 had the lowest TBARS value, 0.13 mg MDA/kg, with no significant (*p* < 0.05) difference compared to that of CFS75. In general, the meat patties with lower fat content had smaller TBARS values. This is consistent with the results of Pan et al., in which meat patties with lower fat levels exhibited better lipid oxidation stability [15]. This phenomenon can be attributed to the fact that meat patties with a lower fat content produce fewer lipid peroxides. In contrast, it was found that the use of a konjac-based oil bulking system to reduce the fat content of meat products led to a significant increase in TBARS values [19], due to the considerable amount of polyunsaturated fatty acids (PUFAs) provided by the oil combination. Other studies have reported using emulsion gels as a fat substitute, which resulted in a decrease in TBARS value and fat content despite the increased proportion of PUFAs in the fat [20,21]. The efficiency of the emulsion gels in protecting the lipid fraction and the low oil content in the system could explain the low lipid oxidation status [6]. In this study, the replacement of pork back fat with CFS was shown to enhance lipid oxidation stability, especially when more than 75% of the fat was replaced.

#### 2.2.3. Color

**Figure 1:**
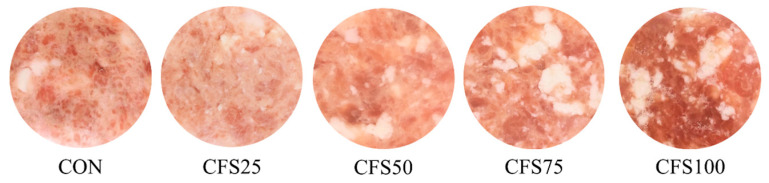
Appearance of fat-replaced patties. The CON are control patties containing only pork fat as a fat resource; CFS25–CFS100 represent 25–100% of pork back fat replaced by cubic fat substitute.

Color is a crucial indicator in the assessment of meat quality, as it can influence consumers’ purchase decisions. The color parameters and appearance of the raw meat patties in this study are provided in Table 2 and Figure 1 separately. In terms of lightness, the CON exhibited the highest L* value. The L* value significantly (*p* < 0.05) decreased when 50% of pork back fat was replaced with CFS but showed no further significant reductions as the replacement ratio increased. The redness (a*) and yellowness (b*) of both CFS25 and CFS50 were not significantly (*p* < 0.05) different from those of the CON; however, the replacement of 75% of the fat with CFS (CFS75) resulted in a significant (*p* < 0.05) increase in both redness and yellowness. Similarly, replacing 40% of fat with CFS was reported to cause an increase in the redness and yellowness of unfermented Harbin dry sausages and a decrease in the lightness of fermented sausages [10]. Pork fat droplets in the matrix of meat dilute the oxymyoglobin and influence the light reflection, thus reducing redness and increasing lightness. Here, the increase in the b* value was attributed to the SPI and coconut oil, both of which imparted a pale-yellow color to the CFS [11]. Different results were reported by Barros et al. after the replacement of 100% of the beef fat in beef burgers with tiger nut oil emulsion, which increased lightness and yellowness but had no significant (*p* < 0.05) impact on the redness of the meat product [22]. An increase in lightness can be attributed to smaller oil globules, which provide greater light reflection. Holman et al. reported that redness can effectively enhance consumer acceptance of beef products since the color is associated with the freshness and value of meat [23]. Consumers tend to prefer lean beef with a bright red color. In this study, higher a* values were observed when replacing more than 75% of fat with CFS, and these patties may be more appealing to consumers.

#### 2.2.4. Technological Characteristics

The replacement of pork back fat with CFS significantly (*p* < 0.05) affected the cooking loss, shrinkage, and moisture retention of the patties (Table 2). As the fat replacement ratio increased, cooking loss and shrinkage decreased in the patties, while moisture retention increased. Cooking loss indicates the loss of weight in patties during the cooking process, and it is an important indicator to consider in food processing. This loss is mainly the result of moisture evaporation and the separation of juices (water and fat) during the heat treatment process. Cooking losses in CFS75 and CFS100 were found to be significantly (*p* < 0.05) lower than the loss for the CON, with values of 6.89% and 6.24%, respectively. This result indicates that replacing the pork back fat in patties with CFS hinders water evaporation and protects the juices against separation. The shrinkage of all samples was significantly (*p* < 0.05) lower than that of the CON, and CFS100 had the lowest shrinkage (6.84%). In agreement, Badar et al. found that cooking loss and shrinkage were reduced in buffalo meat patties by using emulsion hydrogels prepared with different plant oils as fat substitutes [24]. Furthermore, in this study, the CON exhibited the lowest moisture retention, significantly (*p* < 0.05) lower than that in the patties with more than 50% of the fat replaced with CFS, indicating that the replacement of pork back fat with CFS can improve water retention in meat patties. The reason for this phenomenon is twofold: First, KGM has a strong water retention capability, which limits the removal of retained and bound water within the gel network of the CFS by heat or external forces [25]; and, second, the CFS filling the meat matrix may contribute by enhancing the water-holding capacity of the meat systems. In terms of pH, all samples were found to be within 5.88–5.99. There were no significant (*p* < 0.05) differences in the pH values of the CON, CFS25, and CFS50; however, CFS75 and CFS100 showed significant (*p* < 0.05) increases in their pH, with values of 5.93 and 5.99, respectively. This is a similar result to that observed with the changes in moisture retention. The pH levels can affect the water-holding capacity of meat systems, with lower pH values indicating greater moisture loss during processing [26]. Different fat substitutes can have varying effects on the pH values of meat products, depending on the components, processing, and replacement ratio of the fat substitutes [26,27,28,29]. In this study, replacing more than 75% of the fat improved the technological characteristics of the patties.

#### 2.2.5. Texture Profile

As shown in Table 3, replacing pork back fat with CFS significantly (*p* < 0.05) affected the texture characteristics of the cooked meat patties. Increases in the fat replacement ratio were found to increase the hardness, springiness, cohesiveness, and chewiness of the patties. The four texture parameters were significantly (*p* < 0.05) higher in CFS25 and CFS50 than they were in the CON, but similar to each other. However, the chewiness of CFS75 increased significantly (*p* < 0.05) compared to that of CFS25 and CFS50, and all four parameters were significantly (*p* < 0.05) higher in CFS100. These results indicate that the replacement of fat with CFS can enhance the texture of meat patties. Moreover, a gel network with higher gel strength was found to have formed in CFS75 and CFS100. Similar texture changes have been reported in other studies. Pan et al. found that reducing fat and increasing lean meat enhanced the hardness, springiness, cohesiveness, and chewiness of pork patties [15]. The use of oleogel and double emulsion gel to replace pork back fat has also been shown to increase the texture parameters of pork patties [30,31]. In contrast, Freire et al. observed a reduction in the texture parameters of beef patties when using emulsion gels as a fat substitute [31]. Salcedo-Sandoval et al. found that konjac gel alone increased the Kramer shear force in pork patties, which was not the case when using an oil bulking system based on konjac gel [19]. This could have been due to the different properties of the different fat substitutes. Plant oils typically have greater fluidity than animal fats, which can result in a softer texture in reformulated products. Substitute systems with more solid properties may help mitigate this effect.

#### 2.2.6. Sensory Attributes

The sensory scores of the patties are presented in Table 3. The results indicated that replacing the fat with CFS did not significantly (*p* < 0.05) affect the aroma, tenderness, or overall acceptability of the cooked meat patties; however, the substitution did influence their appearance and juiciness. The juiciness scores decreased as the replacement ratio increased. Replacing 100% of the pork back fat with CFS caused a significant (*p* < 0.05) decrease in juiciness, but the loss was lower when less than 75% of the fat was replaced. With the increase in the replacement ratio, the appearance scores increased at first but then decreased. CFS50 had the highest appearance score, significantly (*p* < 0.05) higher than those of CFS75 and CFS100, but with no significant (*p* < 0.05) difference compared to CFS25 and the CON. The reason for these decreased appearance scores could be related to the reduction in pork fat, since fat droplets can provide shine and juiciness. In summary, replacing less than 50% of pork back fat with CFS resulted in patties with better sensory scores, while increasing the replacement ratio led to a deterioration in the juiciness of the meat patties. However, the replacements did not significantly (*p* < 0.05) affect overall acceptability, possibly because the meat patties still demonstrated a good aroma and tenderness. This result concurs with another study, in which reformulated low-fat beef patties were considered acceptable to 55% of the panelists, despite their decrease in sensory score [6]. The replacement of fat in patties with various substitutes and at different replacement ratios is likely to have varying effects on the sensory qualities of patties [31,32,33]. The findings here indicate that, for CFS, a 50% replacement would be optimal in the pursuit of good sensory quality. Furthermore, the complete replacement of fat to achieve low fat content was found to be acceptable from a sensory perspective.

#### 2.2.7. Thermo-Rheology Properties

**Figure 2:**
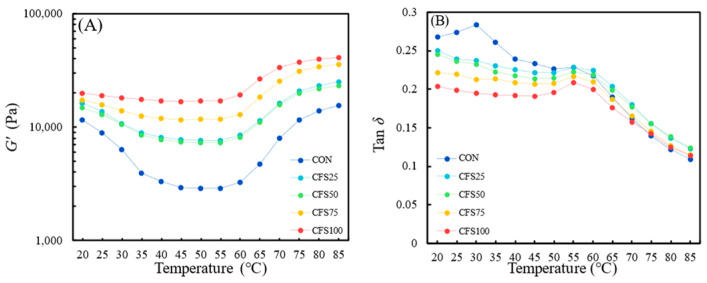
The thermo-rheology curves of fat-replaced patties: (**A**) storage modulus and (**B**) loss factors. CON are the control patties containing only pork fat as a fat resource; CFS25–CFS100 represent 25–100% of pork back fat replaced by cubic fat substitute.

Protein gelation is an important functional property of meat proteins, related to their viscoelasticity and affecting the texture characteristics of meat products [34]. In this work, temperature scanning was used to investigate the thermal gelation of meat protein. The storage modulus (*G*′) was used to measure the elastic behavior of the raw meat batter. The loss factor (tan *δ*) is the ratio of the loss modulus to the storage modulus, and a higher tan *δ* indicates a system with greater fluidity. As shown in Figure 2A, the trend of *G*′ was the same for all samples, decreasing between 20 °C and 45 °C, exhibiting a slight increase at around 50 °C with the appearance of the first peak, and then rising sharply between 55 °C and 85 °C, where it reached its maximum. These results are consistent with those of another study that reported *G*′ trends during the heating of ground pork batter [35]. The changes in *G*′ are related to the denaturation and gelation behavior of muscle proteins. The decrease in *G*′ and the increase in tan δ between 50 °C and 55 °C are due to the denaturation of myosin, which leads to the redistribution of intramolecular and intermolecular forces [36]. Specifically, the denaturation and unfolding of myosin enhance the fluidity of a system. Between 55 °C and 85 °C, exposed functional groups, such as sulfhydryl and hydrophobic groups, undergo cross-linking and aggregation, leading to the formation of a firm, irreversible, and thermal gel, which results in a sharp increase in *G*′ [37]. An increase in the fat replacement ratio results in higher *G*′ in meat batter and indicates that replacing fat with CFS can enhance elasticity in a meat patty and strengthen its thermal gel. This phenomenon could be due to the inherent elasticity of CFS itself, or it could be because the replacement of fat with CFS mitigates the formation of large aggregates of proteins under hydrophobic forces after protein denaturation and, instead, leads to the creation of more cross-linked network structures, resulting in a stronger gel. Pan et al. found that proteins in meat patties with a higher fat content exhibit stronger surface hydrophobicity [38]. Moreover, the KGM in the CFS can enhance the *G*′ and form hydrogen bonds with myosin to mitigate the extent of hydrophobic interactions and protein aggregation, resulting in a dense and uniform network structure [39]. At 20 °C, tan *δ* gradually decreased with the addition of CFS, indicating that higher CFS content will result in a less fluid system. The tan *δ* and *G*′ of the CON were found to be lower than those in the other groups, and *G*′ rapidly decreased between 20 °C and 45 °C, while tan δ increased between 20 °C and 30 °C. This may have been due to the fact that as temperature increases during cooking, semi-solid pig fat transitions into a liquid state, thereby enhancing the fluidity of the system. However, the disparity of tan *δ* among samples decreased during heating. At 85 °C, the tan *δ* of all the samples was at the minimum value and distributed within a narrow range (0.12–0.11), indicating the formation of strong and irreversible thermal gels with similar viscoelasticity. The trend of the *G*′ value at 85 °C is consistent with the texture parameters, both of which showed an increase with the CFS addition. This is in agreement with the SPI addition in the other study, which caused an increase in both the *G*′ and texture parameters of pork patties [35]. Parameters like hardness, Kramer shear force, and *G*′ are usually considered to have a negative correlation with meat tenderness [33]. However, in this study, the tan *δ* value at 85 °C may be more suitable to reflect the tenderness of patties, as the trend of the tenderness score was the same as for tan *δ* instead of being the same as for *G*′ and hardness. This discrepancy may be due to the different mechanisms used to obtain tenderness. Fat may loosen the structure of the meat–protein matrix, reducing hardness and thereby creating a tender perception [15]. Conversely, CFS enhanced the structure and water retention of patties and increased the hardness and *G*′, but had a similar effect to fat in improving tenderness. Another study found that bamboo shoot dietary fiber shows a similar effect, filling in the protein matrix and densifying the structure to retain water, along with an increase in hardness, gel strength, and *G*′ [28]. Moreover, pork patties that had their fat replaced with gelled double emulsions received a lower hardness score in sensory evaluation but had a higher Kramer shear force [31]. Additionally, CFS can also loosen the structure through physical obstruction, decreasing hardness, though not as effectively as fat.

#### 2.2.8. Microstructure

**Figure 3:**
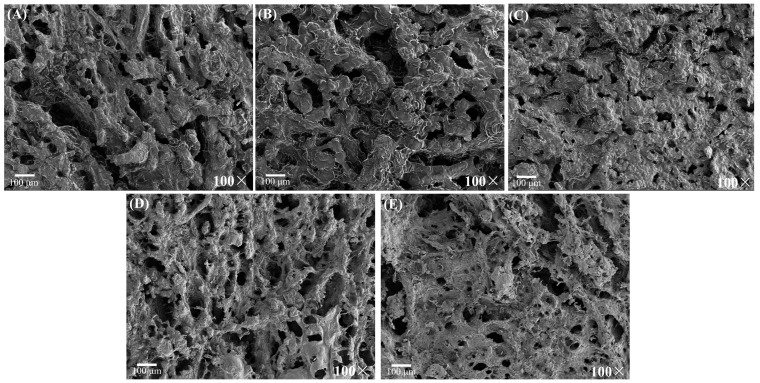
The scanning electron microscopy images of pork patties with different fat replacement levels: (**A**) CON, (**B**) CFS25, (**C**) CFS50, (**D**) CFS75, and (**E**) CFS100. CON are the control patties containing only pork fat as a fat resource; CFS25–CFS100 represent 25–100% of pork back fat replaced by cubic fat substitute.

**Figure 4:**
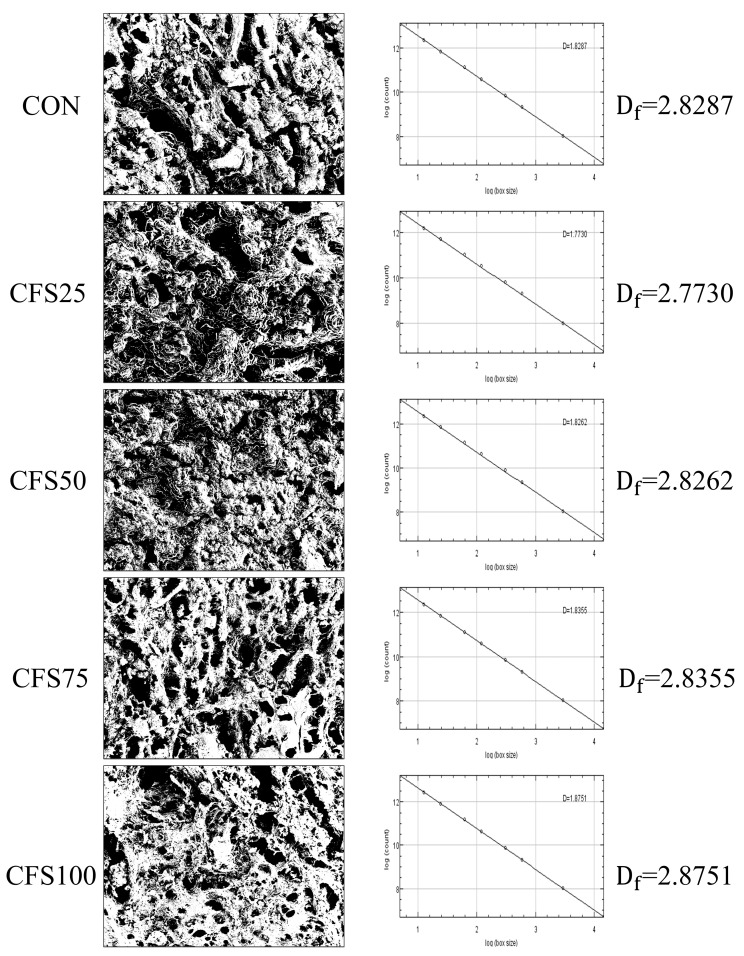
The binary images and fractal dimension values of the microstructures of the fat-replaced patties. CON are the control patties containing only pork fat as a fat resource; CFS25–CFS100 represent 25–100% of pork back fat replaced by cubic fat substitute.

The SEM images (Figure 3) and binary images (Figure 4) illustrate the microstructures of the cooked meat patties. The pores and shadow areas are black in the binary images. The images confirmed that patties with different fat replacement ratios exhibited different network structures. The CON, CFS25, and CFS50 exhibited a coarse network formed by protein aggregation and cross-linked; however, CFS75 and CFS100 exhibited a thready network structure, which is known to provide greater strength, consistent with the texture and rheology results [40]. The meat patties in which fat had been completely replaced by CFS exhibited a smooth laminar structure in their network, formed by the CFS filling within the gel network of the meat–protein matrix. These changes in structure may explain the higher level of moisture retention in meat patties with a high fat replacement ratio. Fractal dimension (*D_f_*) was used to assess the changes in the microstructure of meat patties with different fat replacement ratios. Gel structures with higher *D_f_* values are considered denser, while lower *D_f_* values indicate looser gel networks and a greater number of pores [41]. The *D_f_* values for all images fell within the range of 2.7730 to 2.8751, which is approximately consistent with the range reported in previous research [42]. The results showed that, with an increase in the fat replacement ratio, the *D_f_* values initially decreased and then increased. Specifically, the *D_f_* values for CFS25 and CFS50 were lower than the value for the CON, which was in contrast with the trends observed in the texture and storage moduli. This could have been related to the presence of protein aggregates stacked on the surface, resulting in more shadows, as their pores were smaller than those of the CON. As the fat replacement ratio increased above 50%, the *D_f_* value also increased to more than that of the CON. This is consistent with the result that when the fat replacement ratio was above 75%, there was a reduction in protein aggregates, leading to the formation of a fine and compact cross-linked gel structure in the meat patties.

## 3. Conclusions

In this study, when replacing pork back fat in pork patties with CFS, both the addition of KGM in the CFS and the fat replacement ratio were found to influence the structure and quality of the meat products. Adding 7.0% KGM to CFS reduced the hardness of the reformulated lean meat patties and increased their sensory score. The fat content could be reduced effectively in pork patties by replacing pork back fat with CFS. Moreover, lipid oxidation stability, color, and technological characteristics were all improved as the fat replacement ratio increased. The overall acceptability of the patties was not significantly (*p* < 0.05) influenced by replacing the fat with CFS; however, a deterioration in juiciness was noted. When more than 75% of fat was replaced with CFS, the protein aggregates were reduced and a fine cross-linked gel structure formed, where the smooth CFS paste filled. These results conclusively demonstrated that the CFS prepared by SPI–KGM composite emulsion gels is feasible to partially or completely replace the pork back fat in the pork patties labeled as ‘reduced fat content’. Further research should focus on improving the juiciness of CFS. In addition, the adhesion agents between meat and plant-based gel should be considered. This study provides a theoretical foundation for the application and development of plant-based CFS.

## 4. Materials and Methods

### 4.1. Materials

Fresh pork loin and back fat were purchased from a Freshippo supermarket (Beijing, China) on the day of the patty preparation. The materials used to prepare the CFS were SPI (food grade, protein 91%, Yuwang Ecological Food Industry Co., Ltd., Yucheng, China), KGM (food grade, glucomannan 90%, Yizhi Konjac Biotechnology Co., Ltd., Yichang, China), and coconut oil (food grade, Yuanzhuo Biotechnology Co., Ltd., Xingtai, China). Salt and eggs were purchased from a local supermarket. The other chemical reagents were all of analytical grade (Macklin, Shanghai, China).

### 4.2. Preparation of CFS

The SPI (25 g) was dispersed evenly in distilled water (0–5 °C) and mixed well in a Thermomix food processor (TM-5, Vorwerk, Wuppertal, Germany; set on speed 5) for 4 min. Melted coconut oil (50 g) was then added to the Thermomix, and the solution was emulsified on speed 8 for 5 min before the KGM was gently added to the emulsion and stirred at speed 3 for 3 min. This mixture was then poured into a rectangular polyethylene container (11.6 cm × 8.2 cm × 3 cm), sealed in a vacuum packing bag, and degassed at −0.09 MPa for 20 s using a vacuum packing machine (Exelway, DZ-300, Quanzhou Liding Mechanical Equipment Co., Ltd., Quanzhou, China). Finally, after being incubated in a 95 °C water bath for 1 h and then stored at 4 °C for 24 h, the CFS was ready for use.

### 4.3. Preparation of Pork Patties

The pieces of lean meat, back fat, and CFS were chopped separately using the Thermomix on speed 4 for 1 min each and set aside for further use. The formulae for the lean meat patties and fat-replaced patties are provided in Table 4. To make the meat batter, the materials (400 g in total) were mixed using a Thermomix set on reverse rotation at speed 2 for 1 min. The mixtures were then pressed and shaped into four patties, about 80 g each, 8 cm in diameter, and 2 cm thick. They were subjected to different analyses. The production of patties was carried out three times. All the prepared patties were vacuum packaged and stored at −18 °C for a maximum of 7 days until required for analysis.

### 4.4. Sensory Evaluation

For the sensory evaluation, the raw patties were thawed at room temperature (26 °C) for 2.5 h, then cooked for 12 min at 180 ± 4 °C using a contact grill (MG38CB-AA multi-function electric oven, Midea, Midea Group Co., Ltd., Foshan, China) before being promptly presented to the panelists. The cooked patties for each treatment were evaluated by a panel of 10 students who were trained with experience in the Difference Test and had a consensus on the evaluation content for each attribute (5 males and 5 females, aged between 20 and 30). They were recruited from the College of Food and Health at Beijing Technology and Business University. The whole patties were initially present to score the appearance and flavor, then cut into square pieces for tasting. The 9-point hedonic scale (ranging from 1 = dislike extremely to 9 = like extremely) was used in their assessment of the differently constituted patties in terms of attributes including appearance, juiciness, flavor, tenderness, and overall acceptability. The panelists were asked to clean their mouths with water between tastings to avoid contamination between samples. The presented scores were the average scores received from the 10 panelists. The sensory evaluation was carried out at a constant temperature (26 °C) in a sensory laboratory with permission from the Ethics Review Committee for Scientific Research of Beijing Technology and Business University (IRB-2023-19).

### 4.5. Texture Profile Analysis

For the texture profile analysis (TPA), the cooked patties were cooled for 30 min to room temperature and then cut into cubes (2 cm × 2 cm × 1.5 cm). A texture analyzer (CT3, Brookfield, Middleborough, MA, USA) equipped with a TA4 probe (diameter 38.1 mm) was used to conduct the TPA. Samples were uniaxially compressed twice at a speed of 1 mm/s, with a 5 s pause between the compression cycles and a trigger load of 5 g, until they were 50% of their original height. The load-time variation was recorded using the instrument’s built-in software (TexturePro CT V1.8 Build 31). The measurements were carried out with six replicated cubes from each patty. Hardness was defined as the maximum force of the first compression cycle; springiness as the ratio of the height recovered by the sample after the first compression cycle to the height of its deformation; cohesiveness as the ratio of the curve area during the second compression cycle to the same area during the first cycle; and chewiness was calculated as hardness × springiness × cohesiveness.

### 4.6. Proximate Composition, Color, and Technological Characteristics

The proximate composition (moisture, protein, and fat) and pH value of the raw pork batters were analyzed using the Chinese standard methods GB 2016-5009.3, GB 2016-5009.5, GB 2016-5009.6, and GB 2016-5009.237 [43]. The measurements were carried out in triplicate. The color parameters (L*, a*, and b*) of the raw patties were measured using a CM-3610A colorimeter (Konica Minolta, Tokyo, Japan). During color measurement, the instrument fully covered each frozen meat patty and took measurements at six different positions on its surface. Cooking weight loss, shrinkage, and moisture retention were analyzed using the method described by Tabarestani et al. [44]. The cooking process was performed in triplicate. The measurements were carried out in triplicate.

### 4.7. Lipid Oxidation Stability

TBARS values were measured immediately after the preparation of each raw patty and then measured again following storage in a self-sealing polyethylene bag at 4 °C for 7 days. The method was adapted from the work of John et al., with modifications [45]. The specific steps involved in TBARS measurement were as follows: A 2.00 g sample of meat patty was added to 10 mL of trichloroacetic acid (TCA) solution (7.5% TCA, 0.1% EDTA), then filtered after 10 min of ultrasound extraction at 30 °C. Next, 750 μL of the filtered solution was added to 750 μL of TBARS solution (0.02 mol/L), and the mixture was incubated in a 95 °C water bath for 15 min, then promptly cooled in an ice-water bath. After centrifugation for 5 min at 4 °C and 4000 rpm, the supernatant was collected and its absorbance measured at 532 nm (Cary-60, Agilent, Santa Clara, CA, USA). The measurements were carried out in triplicate. TBARS values (expressed as equivalents of malondialdehyde in the sample, mg MA/kg) were calculated using Formula (1):

(1) $$TBARS=\frac{A\times V}{m}\times 0.462$$

where A is the absorbance; V is the volume of the system (mL); and m is the weight of the sample (g).

### 4.8. Thermo-Rheology Analysis

The thermo-rheological behavior of the raw meat batter was assessed using a stress-controlled rheometer (DHR-1, TA Instruments, New Castle, DE, USA) equipped with a Peltier temperature control device. Temperature scanning was conducted using the method described by Huang et al. [11]. Temperature scanning conditions were as follows: frequency 10 rad/s, strain 1%, temperature range 20–85 °C, heating rate 5 °C/min. The measurements were carried out in triplicate.

### 4.9. Microstructure Observation

The cooked patties were cut into appropriately small pieces and pre-frozen at −40 °C for 48 h, after which the frozen samples were freeze-dried (Beta 1–8 LSC basic, Christ, Osterode, Germany) for 72 h until completely dry. The samples were affixed to a double-sided conductive copper sample holder using tape, and their surfaces were sprayed with gold. Microstructure observation was conducted using a scanning electron microscope (SEM) with an acceleration voltage of 1.5 kV and a secondary electron source. Representative surface morphological characteristics were selected for imaging.

Following the method described by Liu et al. [46], SEM images were processed and analyzed using the public software ImageJ v1.58j8. First, SEM images (2048 × 1775 pixels) were converted into 8-bit binary images with a black background. The fractal dimension (*D_f_*) of each binary image was then calculated using the box-counting method, with box sizes set at 2, 3, 4, 6, 8, 12, 16, 32, and 64.

### 4.10. Statistical Analysis

The reported values correspond to the mean value ± standard deviation. A one-way analysis of variance (ANOVA) with the Tukey test was used to analyze statistical differences among the groups (*p* < 0.05). SPSS 24.0 (IBM Corp., New York, NY, USA) and Excel 2019 (Microsoft, Redmond, Washington, DC, USA) were used to process and analyze the data.

## Figures and Tables

**Table 1:** The sensory score and texture parameters of reformulated lean meat patties.

**Parameters**	**TLP**	**KEG0**	**KEG3.5**	**KEG7**	**KEG10.5**
Sensory characteristics					
Appearance	6.0 ± 0.6 a	4.5 ± 0.8 b	6.0 ± 0.7 a	6.2 ± 0.8 a	4.0 ± 0.9 b
Aroma	5.3 ± 0.8 b	6.3 ± 0.8 a	6.5± 0.5 a	6.3 ± 0.5 a	5.3 ± 0.8 b
Juiciness	6.0 ± 0.7 b	7.1 ± 0.5 a	6.1 ± 0.3 b	6.5 ± 1.1 ab	6.0 ± 0.7 b
Tenderness	5.7 ± 0.5 c	6.7 ± 0.5 ab	6.5 ± 0.5 b	7.1 ± 0.6 a	5.7 ± 0.8 c
Overall acceptability	5.5 ± 0.5 c	6.4 ± 0.5 b	6.7 ± 0.5 ab	7.25 ± 0.5 a	4.4 ± 0.5 d
Texture parameters					
Hardness (N)	89.23 ± 3.32 a	50.20 ± 3.53 b	40.34 ± 3.80 c	25.00 ± 1.99 e	32.82 ± 1.83 d
Springiness	0.79 ± 0.04 ab	0.80 ± 0.04 a	0.72 ± 0.03 bc	0.73 ± 0.05 abc	0.71 ± 0.03 c
Cohesiveness	0.54 ± 0.06 a	0.54 ± 0.03 a	0.40 ± 0.02 bc	0.45 ± 0.02 abc	0.43 ± 0.03 b
Chewiness (N)	36.15 ± 1.78 a	19.24 ± 1.07 b	12.31 ± 0.43 c	13.13 ± 0.30 c	10.24 ± 0.97 c

Note: TLP are totally lean meat patty; KEG0, KEG3.5, KEG7, KEG10.5 represent patties reformulated with konjac emulsion gel containing 0, 3.5, 7,10.5% konjac glucomannan respectively; the same lowercase letters in the rows mean the difference is not significant when using the Tukey test (*p* > 0.05).

**Table 2:** The quality parameters of fat-replaced patties.

Parameters	CON	CFS25	CFS50	CFS75	CFS100
Proximate composition (g/100 g)					
Moisture	58.33 ± 0.82 e	63.05 ± 0.48 d	66.74 ± 0.65 c	70.29 ± 0.13 b	73.13 ± 0.11 a
Protein	17.78 ± 0.12 b	17.79 ± 0.15 b	17.83 ± 0.13 b	18.35 ± 0.10 a	18.41 ± 0.11 a
Fat	21.04 ± 0.46 a	15.62 ± 0.77 b	13.09 ± 0.71 c	6.97 ± 0.09 d	3.65 ± 0.12 e
Fat reduction (%)	-	25.76	37.78	66.87	82.65
Technological characteristics					
pH value	5.89 ± 0.02 b	5.88 ± 0.02 b	5.89 ± 0.00 b	5.93 ± 0.00 a	5.99 ± 0.02 a
Cooking loss (%)	11.75 ± 1.10 b	10.92 ± 1.38 b	8.53 ± 1.50 ab	6.89 ± 1.98 a	6.24 ± 0.29 a
Shrinkage (%)	19.75 ± 1.77 a	14.47 ± 0.37 b	11.50 ± 1.41 bc	8.55 ± 0.60 cd	6.84 ± 0.74 d
Water retention (%)	83.26 ± 1.96 c	84.25 ± 0.59 c	85.59 ± 1.02 bc	88.71 ± 2.02 ba	90.35 ± 0.54 a
TBARS value (mg MDA/kg sample)					
0 d	0.06 ± 0.01 a	0.06 ± 0.01 a	0.07 ± 0.01 a	0.07 ± 0.02 a	0.05 ± 0.01 a
7 d	0.38 ± 0.02 a	0.25 ± 0.01 b	0.21 ± 0.01 c	0.16 ± 0.01 d	0.13 ± 0.01 d
Color parameters					
L*	68.93 ± 0.12 a	65.92 ± 1.69 a	61.12 ± 1.04 b	58.89 ± 1.37 b	59.51 ± 1.14 b
a*	9.53 ± 0.03 b	9.36 ± 0.63 b	10.48 ± 0.35 ab	11.82 ± 0.86 a	12.23 ± 0.88 a
b*	22.07 ± 0.30 bc	20.65 ± 0.94 c	22.84 ± 1.15 abc	24.44 ± 1.18 ab	25.91 ± 0.92 a

Note: CON are control patties containing only pork fat as a fat resource; CFS25–CFS100 represent 25–100% of pork back fat replaced by cubic fat substitutes; the same lowercase letters in the rows mean the difference is not significant when using the Tukey test (*p* > 0.05).

**Table 3:** The sensory scores and texture parameters of fat-replaced patties.

Parameters	CON	CFS25	CFS50	CFS75	CFS100
Sensory characteristics					
Appearance	5.4 ± 1.1 b	6.5 ± 0.8 a	6.7 ± 0.5 a	5.4 ± 0.7 b	5.4 ± 0.8 b
Aroma	5.3 ± 0.7 a	5.4 ± 0.5 a	5.5 ± 0.7 a	5.3 ± 0.9 a	5.2 ± 0.6 a
Juiciness	7.0 ± 1.1 a	7.2 ± 0.6 a	6.6 ± 0.8 ab	6.2 ± 0.6 ab	5.7 ± 0.7 b
Tenderness	6.3 ± 0.8 a	7.0 ± 0.5 a	6.4 ± 0.7 a	6.7 ± 1.1 a	6.6 ± 1.1 a
Overall acceptability	6.6 ± 1.0 a	6.9 ± 0.9 a	6.9 ± 1.0 a	6.5 ± 0.7 a	6.4 ± 0.7 a
Texture parameters					
Hardness (N)	13.97± 0.82 c	18.39 ± 0.08 b	17.70 ± 0.55 b	18.81 ± 0.47 b	23.67 ± 1.94 a
Springiness	0.44 ± 0.01 d	0.53 ± 0.02 c	0.56 ± 0.04 bc	0.61 ± 0.03 b	0.69 ± 0.03 a
Cohesiveness	0.24 ± 0.02 c	0.28 ± 0.01 b	0.27 ± 0.00 b	0.29 ± 0.02 b	0.34 ± 0.02 a
Chewiness (N)	1.16 ± 0.04 d	2.94 ± 0.18 c	2.81 ± 0.16 c	3.40 ± 0.31 b	6.17 ± 0.14 a

Note: CON are the control patties containing only pork fat as a fat resource; CFS25–CFS100 represent 25–100% of pork back fat replaced by cubic fat substitute; the same lowercase letters in the rows mean the difference is not significant when using the Tukey test (*p* > 0.05).

**Table 4:** The formulae of CFS and meat patties.

Ingredients (g/100 g)	Lean Meat Patties					Fat-Replaced Patties				
	TLP	KEG0	KEG3.5	KEG7	KEG10	CON	CFS25	CFS50	CFS75	CFS100
CFS										
SPI	-	5	5	5	5	5	5	5	5	5
Coconut oil	-	10	10	10	10	10	10	10	10	10
KGM	-	0	3.5	7	10.5	7	7	7	7	7
Water	-	85	81.5	78	74.5	78	78	78	78	78
Meat patties										
Lean meat	94	70	70	70	70	70	70	70	70	70
Pork back fat	-	-	-	-	-	24	18	12	6	0
CFS	0	24	24	24	24	0	6	12	18	24
Egg white	4	4	4	4	4	4	4	4	4	4
Salt	2	2	2	2	2	2	2	2	2	2

## Data Availability

The data presented in this study are openly available in article.
